# Synthetic biology of cyanobacteria: unique challenges and opportunities

**DOI:** 10.3389/fmicb.2013.00246

**Published:** 2013-08-27

**Authors:** Bertram M. Berla, Rajib Saha, Cheryl M. Immethun, Costas D. Maranas, Tae Seok Moon, Himadri B. Pakrasi

**Affiliations:** ^1^Department of Energy, Environmental, and Chemical Engineering, Washington UniversitySt. Louis, MO, USA; ^2^Department of Chemical Engineering, Pennsylvania State University, University ParkPA, USA; ^3^Department of Biology, Washington University in St. LouisSt. Louis, MO, USA

**Keywords:** cyanobacteria, synthetic biology, systems biology, biofuel, flux balance analysis, metabolic flux analysis

## Abstract

Photosynthetic organisms, and especially cyanobacteria, hold great promise as sources of renewably-produced fuels, bulk and specialty chemicals, and nutritional products. Synthetic biology tools can help unlock cyanobacteria's potential for these functions, but unfortunately tool development for these organisms has lagged behind that for *S. cerevisiae* and *E. coli*. While these organisms may in many cases be more difficult to work with as “chassis” strains for synthetic biology than certain heterotrophs, the unique advantages of autotrophs in biotechnology applications as well as the scientific importance of improved understanding of photosynthesis warrant the development of these systems into something akin to a “green *E. coli*.” In this review, we highlight unique challenges and opportunities for development of synthetic biology approaches in cyanobacteria. We review classical and recently developed methods for constructing targeted mutants in various cyanobacterial strains, and offer perspective on what genetic tools might most greatly expand the ability to engineer new functions in such strains. Similarly, we review what genetic parts are most needed for the development of cyanobacterial synthetic biology. Finally, we highlight recent methods to construct genome-scale models of cyanobacterial metabolism and to use those models to measure properties of autotrophic metabolism. Throughout this paper, we discuss some of the unique challenges of a diurnal, autotrophic lifestyle along with how the development of synthetic biology and biotechnology in cyanobacteria must fit within those constraints.

## Introduction

Cyanobacteria have garnered a great deal of attention recently as biofuel-producing organisms. Their key advantage over other bacteria is their ability to use photosynthesis to capture energy from sunlight and convert CO_2_ into products of interest. As compared with eukaryotic algae and plants, cyanobacteria are much easier to manipulate genetically and grow much faster. They have been engineered to produce a wide and ever-expanding range of products including fatty acids, long-chain alcohols, alkanes, ethylene, polyhydroxybutyrate, 2,3-Butanediol, ethanol, and hydrogen. These processes have been reviewed recently (Gronenberg et al., 2013) and will not be covered in detail in this review. Rather, we will look toward how the techniques of the emerging field of synthetic biology might bear fruit in improving the output of such engineered strains. Due to the low price of commodity goods like fuels and platform chemicals, it is critical to maximize the productivity of engineered strains to make them economically competitive. We believe that the tools of synthetic biology can help with this challenge.

Specifically, this review will cover systems, parts, and methods of analysis for synthetic biology. Synthetic biology requires a well-characterized host or “chassis” strain that can be genetically manipulated with ease and predictability. Ideally, the host should grow quickly and tolerate a range of environmental conditions. The host should be simple to cultivate using readily available laboratory equipment and inexpensive growth media. Simple, rapid, and high-throughput techniques should be available for procedures like DNA/RNA isolation, metabolomics, and proteomics. To achieve modular, “plug-and-play” modification of the host strain, its metabolism and regulatory systems must be well-characterized under a wide variety of relevant conditions. Since cyanobacterial biofuel production processes will need to use sunlight as an energy source to be economically and environmentally useful, the day/night cycle will be particularly relevant; the intermittent nature of this energy source will be a key engineering challenge. We will discuss which cyanobacterial chassis have been used and their relative merits and unique traits. Ultimately, the hope is that one of these strains might be developed to become a “green *E. coli*” for which a wide variety of genetic parts and systems are available for easy modification. Next, we will discuss the critical issue of how gene expression can be controlled in cyanobacteria. Compared with other systems, there are few examples of simple and effective controllable promoters in cyanobacteria. We will also discuss methods for analysis of gene expression using light-emitting reporters and for global analysis of metabolism using either constraint-based modeling or measurement of ^13^C labeling.

## Genetic modification of cyanobacteria

Several strains of cyanobacteria are known which are readily amenable to genetic modification (See Table 1). Such modifications can be performed either *in cis* (through chromosome editing) or *in trans* (through plasmid addition) and synthetic biology experiments have used both approaches. We discuss advantages and disadvantages of each approach, as well as recent technical developments below. While even the best cyanobacterial model systems are still far from being a “green *E. coli*,” many tools are already available and more are being developed. The future holds great promise for this field.

**Table 1:** **Model strains of cyanobacteria for synthetic biology**.

**Strain**	**Genetic methods**	**Ideal growth temp (C)**	**Doubling time (h)**	**Metabolisms**	**Genome-scale models?**	**Notes**	**References**
*Synechocystis* sp. PCC 6803	Conjugation, natural transformation, Tn5 mutagenesis, fusion PCR	30	6–12	Mixotrophic, autotrophic	Yes	Extensive systems biology datasets are available	Heidorn et al., 2011
*Synechococcus elongatus* PCC 7942	Conjugation, natural Transformation, Tn5 mutagenesis	38	12–24	Autotrophic	No	A model strain for the study of circadian clocks	Chen et al., 2012
*Synechococcus* sp. PCC 7002	Conjugation, natural transformation	38	3.5	Mixotrophic, autotrophic	Yes	Among the fastest-growing strains known	Xu et al., 2011
*Anabaena variabilis* PCC 7120	Conjugation, natural transformation	30	>24	Mixotrophic, autotrophic	No	Nitrogen-fixing, Filamentous	Zhang et al., 2007
*Leptolyngbya* sp. Strain BL0902	Conjugation, Tn5 mutagenesis	30	~20	Autotrophic	No	Filamentous, Grows well in outdoor photo-bioreactors in a broad range of conditions	Taton et al., 2012

### Genetic modification in Cis: chromosome editing

**Figure 1:**
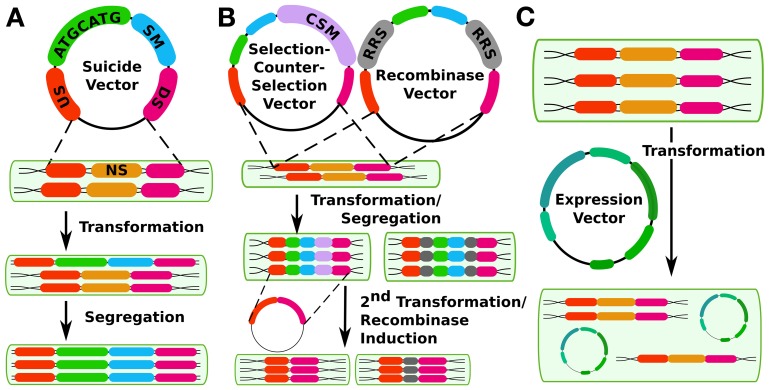
**Different methods for constructing cyanobacterial mutants. (A)** shows the traditional method using double homologous recombination to insert a suicide vector into the genome at a neutral site (NS, gold) with upstream (US, orange) and downstream (DS, magenta) flanking regions in the vector. The insert contains an arbitrary sequence of interest (ATGCATG, green) and a selectable marker (SM, blue). **(B)** shows two methods of creating markerless mutants, either by selection-counterselection or by using a recombinase system such as FLP/FRT, The counter-selection method's first step is the same as for the method in panel a, except that the insert also contains a counter-selectable marker (CSM, purple) such as *sacB*. A second transformation is performed to create a markerless mutant. Alternatively, the insert can contain recombinase recognition sites (RRS, gray) that are controlled by an inducible recombinase at a second (or the same) site in the genome. While it erases the selectable marker, this method does leave a scar sequence behind. **(C)** shows genetic modification *in trans* via expression plasmids.

*Cis* genetic modification is the most common approach in cyanobacterial synthetic biology. This approach takes advantage of the capability of many cyanobacterial strains for natural transformation and homologous recombination (see Table 1) to create insertion, deletion, or replacement mutations in cyanobacterial chromosomes. Traditionally, strains have been transformed with selectable markers linked to any sequence of interest and flanked by sequences homologous to any non-essential sequence on the chromosome (See Figure 1).

This strategy allows the creation of targeted mutations to the chromosome, but sometimes raises concerns about segregation in polyploid strains. However, once segregated, such mutations can be stable over long time periods even in the absence of selective pressure from added antibiotics (Liu et al., 2011; Wang et al., 2013). While such stability is desirable, systems that create major metabolic demand, by for example redirecting flux into biofuel-producing pathways, will face greater selective pressures for mutation or loss of heterologous genes.

Recently, several methods have been developed that allow the creation of markerless mutations in cyanobacterial chromosomes (Figure 1B). Two of these methods operate on a similar principle: First, a conditionally toxic gene is linked to an antibiotic resistance cassette and then inserted into the chromosome, with selection for antibiotic-resistant mutants. Next, a second transformation is carried out in which the resistance cassette and toxin gene are deleted, and markerless mutants are selected which have lost the toxic gene. This principle has been used in cyanobacteria with the *B. subtilis* levansucrase synthase gene *sacB*, which confers sucrose sensitivity (Lagarde et al., 2000) as well as with *E. coli mazF*, a general protein synthesis inhibitor expressed under a nickel-inducible promoter (Cheah et al., 2013). This latter system has advantages for cyanobacterial strains that are naturally sucrose-sensitive. Either method allows the reuse of a single selectable marker for making multiple successive changes to the chromosome. In addition to these methods, a third system operates on a similar principle—a cyanobacterial strain that is streptomycin resistant due to a mutation in the *rps12* gene can be made streptomycin-sensitive by expressing a second heterologous copy of wild type *rps12* linked to a kanamycin (or other antibiotic) resistance cassette as well as any sequence of interest. Streptomycin-resistant, kanamycin-sensitive markerless mutants can be recovered in a second transformation (Takahama et al., 2004). Although this method can also be used to make successive markerless mutants, it requires a background strain that is streptomycin-resistant due to an altered ribosome. Thus, it may not be an ideal method for synthetic biology studies that seek to draw conclusions about translation in wild-type systems. For the ability to transfer any translated genetic parts or parts involved in translation (such as ribosome binding sites) to other strains, this mutation could be problematic. A possible advantage of this system is that both selections are positive selections, whereas the *sacB* or *mazF* systems require a negative selection in their second transformation. Care must be taken to ensure that sucrose resistance is due to loss, as opposed to mutation, of the counter-selectable marker. Recombinase-based systems including Cre-LoxP [in *Anabaena sp.* PCC7120, (Zhang et al., 2007)] or FLP/FRT [in *Synechocystis sp.* PCC6803 and *Synechococcus elongatus* PCC7942, (Tan et al., 2013)] have also been used to engineer mutants that lack a selectable marker. However, these methods leave a scar sequence, meaning that the final chromosomal sequence is not completely user-specifiable and also that multiple mutations using this technique in the same cell line may potentially lead to undesirable crossover events or other unexpected results.

Until recently, it has been difficult to create mutants at high throughput in cyanobacterial strains, as transposon-based methods developed for use in other strains can work poorly in cyanobacterial hosts. However, libraries can be created in other strains and subsequently transferred to a cyanobacterial host via homologous recombination. A Tn7-based library containing ~10,000 lines was recently created to screen for strains with increased polyhydroxybutyrate (PHB) production (Tyo et al., 2009) and a similar approach has been taken for finding mutants in circadian clock function in *Synechococcus* 7942 (Holtman et al., 2005) and later extended to include insertions into nearly 90% of open reading frames in that strain (Chen et al., 2012). Chromosomal DNA fragments were first cloned into a plasmid library in *E. coli* and then the library was mutagenized with Tn7 before homologous recombination back into the cyanobacterial host strain. This could be an especially valuable approach for the validation of genome-scale models of cyanobacterial metabolism (see below).

### Genetic modification *in trans*: foreign plasmids

**Figure 2:**
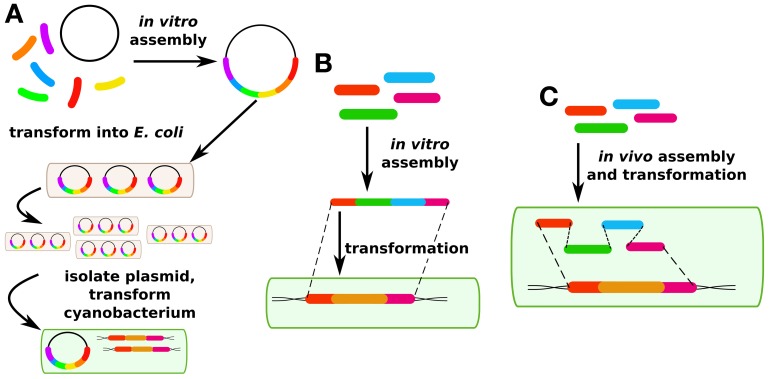
**DNA assembly methods. (A)** Traditionally in cyanobacterial synthetic biology, plasmids are assembled *in vitro* and then propagated in *E. coli* before being transformed into cyanobacteria. **(B)** More recently, methods have been developed for *in vitro* assembly and direct transformation via fusion PCR. **(C)** Another recent method has been developed for *in vivo* plasmid assembly via homologous recombination in yeast which may also be applicable in certain cyanobacterial strains.

Although transgene expression *in cis* is the most common approach in cyanobacterial research, genes are also routinely expressed in cyanobacteria *in trans* (Huang et al., 2010; Landry et al., 2012; Huang and Lindblad, 2013). In synthetic biology and metabolic engineering of other prokaryotes, this is by far the more common approach, and has led to such standardized approaches as “Bio-Brick” assembly in which standardized genetic “parts” such as promoters, ribosome binding sites, genes, and terminators can be readily swapped in and out of standard plasmids (http://partsregistry.org). This move toward standardization of genetic parts is a critical aim for synthetic biology, independent of the chassis organism or method of transformation. However, a limited number of plasmids are available for expression in cyanobacterial hosts. Plasmid assembly for expression *in cis* or *in trans* in cyanobacterial hosts has generally been performed in *E. coli* because of the longer growth times that would be associated with assembling vectors in cyanobacterial hosts (Figure 2A). This requires broad host range plasmids. However, with the rise of *in vitro* assembly methods such as SLIC (Li and Elledge, 2007), Gibson assembly (Gibson et al., 2009), CPEC (Quan and Tian, 2009), fusion PCR (Szewczyk et al., 2007), and Golden Gate (Engler and Marillonnet, 2011), this limitation may become less important over time (Figure 2B). These next-generation cloning methods have been reviewed elsewhere (Hilson et al., 2012) and will not be covered here. Fusion PCR has been used to construct linear DNA fragments for homologous recombination in cyanobacterial chromosomes (Nagarajan et al., 2011), but to our knowledge replicative vectors for cyanobacteria have so far not been constructed without the use of a helper heterotrophic strain. Techniques for *in vivo* assembly of plasmids that have been developed for yeast (Shao and Zhao, 2009) may be adaptable to cyanobacteria because of their facility for homologous recombination (Figure 2C). Such an improvement could greatly speed up the process of making cyanobacterial mutant strains, either for modification *in cis* or *in trans*. The major technical challenge for such an approach is that the long time after transformation required to isolate cyanobacterial mutants (typically 1 week or more) means it is critical to have high-fidelity assembly methods to avoid a time-consuming screening process.

Although shuttle vectors do exist for cyanobacteria, there has been little characterization of their copy numbers in cyanobacterial hosts, and the lack of replicative vectors with varied copy numbers limits the valuable ability to control the expression level of heterologous genes by selecting their copy number (Jones et al., 2000; Dunlop et al., 2011). Plasmids derived from RSF1010 appear to have a copy number of 10–30 (or ~1–3 per chromosome) in *Synechocystis sp.* PCC 6803 (Ng et al., 2000; Huang et al., 2010), but copy numbers of other broad host-range plasmids have not been quantified to date. Endogenous plasmids of cyanobacteria have also been used as target sites for expression of heterologous genes in *Synechococcus sp.* PCC 7002 (Xu et al., 2011). This strain harbors several endogenous plasmids whose copy numbers range from ~1 to 8 per chromosome, with an approximate chromosome copy number of 6 per cell. *Synechocystis* sp. PCC 6803 also has plasmids whose copy numbers span a similar range [from ~0.4–8 per chromosome (Berla and Pakrasi, 2012)]. The origins of replication from these plasmids constitute a source of genetic parts that could be used to generate cyanobacterial expression plasmids having a range of copy numbers, and which could potentially be modified to create higher or lower-copy plasmids that are compatible with existing plasmids in various cyanobacterial systems. The range of shuttle vectors that have been used in cyanobacterial hosts has been recently reviewed (Wang et al., 2012). While many tools are available for genetic modification of these biotechnologically promising strains, opportunities abound to develop new and improved tools that will allow research to proceed faster.

## Unique challenges of the cyanobacterial lifestyle

Organisms that survive using sunlight as a primary nutrient face unique challenges. These must be better understood and addressed to fulfill the biotechnological promise of cyanobacteria through synthetic biology.

### Life in a diurnal environment

A primary goal of synthetic biology in cyanobacteria is to use photosynthesis to convert CO_2_ into higher-value products such as biofuels and chemical precursors. To make such a process economically and environmentally feasible will require using sunlight as a primary energy source. While some cyanobacteria are facultative heterotrophs, their key advantage over obligate heterotrophic bacteria is photosynthesis. Unlike heterotrophic growth environments where carbon and energy sources can be provided more uniformly both in space and time, sunlight will only be available during the day and will be attenuated as it passes through the culture. Under certain conditions, cultures may be able to take advantage of a “flashing light effect” to integrate spatially uneven illumination by storing chemical energy when in bright light near the reactor surface and using that energy to conduct biochemistry during time spent in the dark away from the reactor surface. This ability will depend on light intensity, mixing rates, reactor geometry, and likely other factors. Certain diazotrophic cyanobacteria can even use daylight to continue growth during the night. *Cyanothece sp*. ATCC 51142 [along with several other strains (Taniuchi et al., 2008; Latysheva et al., 2012; Pfreundt et al., 2012)] is a unicellular diazotrophic cyanobacterium that performs photosynthesis and accumulates glycogen during the day, and then during the night breaks down its glycogen reserves to supply energy for nitrogen fixation. Thus, these strains spread out the energy available from sunlight over a 24-h period. This process involves a genome-wide oscillation in transcription, with more than 30% of genes oscillating in expression between day and night (Stockel et al., 2008). To take full advantage of sunlight, synthetic systems must be created that are capable of responding appropriately to this challenging dynamic environment. It has recently been shown that biofuel-producing strains that dynamically tune the expression of heterologous pathways in response to their own intracellular conditions produce more biofuel and exhibit greater stability of heterologous pathways (Zhang et al., 2012). As challenging as the design of such a system was for batch heterotrophic cultures, it will be even more challenging in production environments that include a diurnal light cycle.

While not all strains exhibit as complete a physiological change between day and night as *Cyanothece* 51142, all cyanobacteria do have a circadian clock that adapts them to their autotrophic lifestyle. The cyanobacterial circadian clock is anchored by master regulators KaiA, KaiB, and KaiC, which act by cyclically phosphorylating and dephosphorylating each other (Akiyama, 2012). While the circadian rhythm can be reconstituted *in vitro* using the three Kai proteins in the presence of ATP (Nakajima et al., 2005), the accurate maintenance of this clock *in vivo* depends on proper protein turnover (Holtman et al., 2005), on codon selection in the *kaiBC* transcript (Xu et al., 2013), on transcriptional feedback (Teng et al., 2013), and on the controlled response of the entire program of cellular transcription to the output of the KaiABC oscillator. While disturbing rhythmicity can lead to strains that grow better under constant light, the circadian clock is adaptive for strains living in a dynamic environment (Woelfle et al., 2004; Xu et al., 2013). Therefore, integrating synthetic gene circuits such as biofuel production processes into the circadian rhythm of cyanobacterial hosts will likely lead to both improved production and improved strain stability in outdoor production environments.

### Redirecting carbon flux by decoupling growth from production

While redirecting carbon flux is a challenge in all metabolic engineering efforts, it has been suggested that stringent control of fixed carbon partitioning among central metabolic pathways poses a major limitation to chemical production especially in photosynthetic organisms (Melis, 2013). During the growth phase, it may be true that carbon partitioning is tightly controlled by any number of mechanisms including metabolite channeling or simply high demand for metabolic intermediates. However, biofuel production during non-growth phases (Atsumi et al., 2009; Liu et al., 2011; Wang et al., 2013) demonstrates that under appropriate conditions, cyanobacterial hosts can produce biofuel compounds with higher selectivity, since biofuel can be produced by metabolically active cells even in the absence of growth. Enhancing their productivity in this phase is a major opportunity for cyanobacterial synthetic biologists to overcome these limits on carbon partitioning. Capturing this opportunity will require designing complete metabolic circuits that remain highly active during stationary phase.

### RNA-based regulation

Recently, regulation of gene expression through RNA mechanisms has received great attention across bacterial clades (Selinger et al., 2000; Sharma et al., 2010; Mitschke et al., 2011). While these mechanisms of regulation may be important in all bacteria, their prominence is perhaps the greatest in the cyanobacteria and may help these diurnal organisms adapt to their highly dynamic environment: in a recent dRNA-seq study, many of the most highly expressed RNAs belonged to families of non-coding RNAs which are present in nearly all sequenced cyanobacteria, but not in any other organisms (Gierga et al., 2009; Mitschke et al., 2011). While their high expression in *Synechocystis* 6803 suggests functional importance for non-coding RNAs, few have clearly elucidated functions to date. *syr1* overexpression has been shown to lead to a severe growth defect in *Synechocystis* 6803 (Mitschke et al., 2011). Another small RNA, *isiR*, has a critical function in stress response in *Synechocystis* 6803. *isiR* binds to the mRNA (*isiA*) for the iron-stress inducible protein, which when translated, forms a ring around trimers of photosystem I, preventing their activity and thus oxidative stress in the absence of sufficient iron (Duhring et al., 2006). The binding of *isiR* to *isiA* appears to result in rapid degradation. This particular arrangement allows a very rapid and emphatic response to iron repletion in cyanobacteria, since a large pool of *isiA* transcripts can be quickly silenced and marked for degradation by transcription of the antisense *isiR*. Although little is so far known about the generality of this type of regulation, the dynamics of this response might also be effective to use for synthetic systems in cyanobacteria that live in the presence of light as an intermittently available but critical nutrient.

While non-coding RNA has received a lot of recent attention, two-component systems make up the most widely studied family of environmental response regulators in cyanobacteria. Many of these systems have known functions in response to diverse environmental stimuli such as nitrogen, phosphorous, CO_2_, temperature, salt, and light intensity and quality (Ashby and Houmard, 2006; Montgomery, 2007). Many of the most widely-used systems in the construction of synthetic biological devices (such as the *ara* and *lux* clusters) use 2-component systems, and even combine 2-component systems with non-coding RNA to control system dynamics (Waters and Bassler, 2006). As synthetic biology advances into the construction of more and more complex systems, there will be a growing need to understand and use all of the different mechanisms available for control of gene expression and enzyme activity in cyanobacteria.

## Parts for cyanobacterial synthetic biology

While cyanobacteria are promising organisms for biotechnology, synthetic biology tools for these organisms lag behind what has been developed for *E. coli* and yeast (Heidorn et al., 2011). Furthermore, synthetic biology tools developed in *E. coli* or yeast often do not function as designed in cyanobacteria (Huang et al., 2010). Here, we discuss inducible promoters and reporters in cyanobacteria, and cultivation systems that will allow their testing at increased throughput. Refining such systems will make cyanobacterial synthetic biology more user-friendly, a central goal for developing the “green *E. coli*.”

### Inducible promoters

Creation of synthetic biology systems that predictably respond to a specific signal often depends upon inducible promoters for transcriptional control. An ideal inducible promoter will have the following properties: (1) It will not be activated in the absence of inducer. (2) It will produce a predictable response to a given concentration of inducer or repressor. This response may be digital (i.e., on/off) or graded change with different concentrations of inducer/repressor. (3) The inducer at saturating concentrations should have no harmful effect on the host organism. (4) The inducer should be cheap and stable under the growth conditions of the host. Finally, (5) the inducible system should act orthogonally to the host cell's transcriptional program. Ideal transcriptional repressors should not bind to native promoters and if non-native transcriptional machinery is used (such as T7 RNA polymerase) it should not initiate transcription from native promoters. Promoters must perform as ideally as possible in order to be used in the construction of more complex genetic circuits (Moon et al., 2012).

Many common inducible promoters in cyanobacteria respond to transition metals. These have often been the basis of metal detection systems (Erbe et al., 1996; Boyanapalli et al., 2007; Peca et al., 2007, 2008; Blasi et al., 2012). Cyanobacteria balance metal intake for the organisms' needs against potential oxidative stress and protein denaturation (Michel et al., 2001; Peca et al., 2008) via tightly regulated systems. As shown in Table 2, cyanobacteria's metal-responsive promoters frequently show greater than 100-fold dynamic range. For example, the promoter for the *Synechocystis* sp. PCC 6803 gene, *coaA*, was induced 500-fold by 6 μ M Co^2+^ (Guerrero et al., 2012), and *P*_*smt*_ from *Synechococcus elongatus* PCC 7942 was induced 300-fold by 2 μ M Zn^2+^ (Erbe et al., 1996). The most responsive cyanobacterial promoters reported were P*_nrsB_* from *Synechocystis* sp. PCC 6803, responding 1000-fold to 0.5 μ M Ni^2+^(Peca et al., 2007), and P*_isiAB_* also from *Synechocystis* sp. PCC 6803, repressed 5000-fold by 30 μ M Fe^3+^ following depletion (Kunert et al., 2003).

**Table 2:** **Inducible promoters used in cyanobacterial hosts**.

**Promoter**	**Source**	**Inducer/repressor and concentration**	**Expression host**	**Expressed gene**	**Dynamic range**	**Measure of expression**	**References**
**METAL-INDUCIBLE PROMOTERS**							
*ArsB*	*Synechocystis* sp. PCC 6803	Inducer AsO^2−^ 720 Mm	*Synechocystis* sp. PCC 6803	*arsB*	100-fold	RT-PCR	Blasi et al., 2012
*ZiaA*	*Synechocystis* sp. PCC 6803	Inducer Cd^2+^ 2 μ M	*Synechocystis* sp. PCC 6803	*ziaA*	10-fold	RT-PCR	Blasi et al., 2012
*coat*	*Synechocystis* sp. PCC 6803	Inducer Co^2+^ 6 μ M	*Synechocystis* sp. PCC 6803	Gene encoding EFE from *Pseudomonas syringae*	500-fold	48 nL ethylene mL^−1^ h^−1^	Guerrero et al., 2012
*coat*	*Synechocystis* sp. PCC 6803	Inducer Co^2+^ 6.4 μ M	*Synechocystis* sp. PCC 6803	*coaR + luxAB*	70-fold	70 RLU^e^	Peca et al., 2008
*coat*	*Synechocystis* sp. PCC 6803	Inducer Co^2+^ 3 μ M	*Synechocystis* sp. PCC 6803	*coaT*	10-fold	RT-PCR	Peca et al., 2007
*nrsB*	*Synechocystis* sp. PCC 6803	Inducer Co^2+^ 3 μ M	*Synechocystis* sp. PCC 6803	*nrsB*	10-fold	RT-PCR	Peca et al., 2007
*coat*	*Synechocystis* sp. PCC 6803	Inducer Co^2+^ 1 μ M	*Synechocystis* sp. PCC 6803	*coaT*	10-fold	RT-PCR	Blasi et al., 2012
*petE*	*Synechocystis* sp. PCC 6803	Inducer Cu^2+^ 0.5 μ M	*Synechocystis* sp. PCC 6803	Gene encoding EFE from *Pseudomonas syringae*	5-fold	28 nL ethylene mL^−1^ h^−1^	Guerrero et al., 2012
*petE*	*Synechocystis* sp. PCC 6803	Inducer Cu^2+^ 3 μ M	*Anabaena* sp. PCC 7120	*hetP*	4.5-fold	8% heterocyst frequency	Higa and Callahan, 2010
*petE*	*Synechocystis* sp. PCC 6803	Inducer Cu^2+^ 0.3 μ M	*Anabaena* sp. PCC 7120	*hetN* (prevents heterocyst formation)	Qualified but not quantified	0% heterocysts from 10% uninduced	Callahan and Buikema, 2001
*isiAB*	*Synechocystis* sp. PCC 6803	Repressor Fe^3+^ 30 μ M	*Synechocystis* sp. PCC 6803	*isiAB + gfp*	5000-fold	From 5000 RFU^e^	Kunert et al., 2003
*idiA*	*Synechococcus elongatus* PCC 7942	Repressor Fe^2+^ 0.043 mM	*Synechococcus elongatus* PCC 7942	*luxAB*	170-fold	Luminescence (5.3 × 10^6^ cpm)	Michel et al., 2001
*isiAB*	*Synechococcus* sp. strain PCC 7002	Repressor Fe^3+^ 100 nM	*Synechococcus* sp. strain PCC 7002	*luxAB from Vibrio harveyi*	2-fold	From 0.012 RLU cell^−1^ s^−1^	Boyanapalli et al., 2007
*nrsB*	*Synechocystis* sp. PCC 6803	Inducer Ni^2+^ 0.5 μ M	*Synechocystis* sp. PCC 6803	*nrsB*	1000-fold	RT-PCR	Peca et al., 2007
*nrsB*	*Synechocystis* sp. PCC 6803	Inducer Ni^2+^ 5 μ M	*Synechocystis* sp. PCC 6803	*nrsB*	400-fold	RT-PCR	Blasi et al., 2012
*nrsB*	*Synechocystis* sp. PCC 6803	Inducer Ni^2+^ 6.4 μ M	*Synechocystis* sp. PCC 6803	*nrsR + luxAB*	50-fold	50 RLU^e^	Peca et al., 2008
*Smt*	*Synechococcus elongatus* PCC 7942	Inducer Zn^2+^ 2 μ M	*Synechococcus elongatus* PCC 7942	*luxCDABE* from *Vibrio fisheri*	300-fold	325,000 cps luminescence	Erbe et al., 1996
*ziaA*	*Synechocystis* sp. PCC 6803	Inducer Zn^2+^ 5 μ M	*Synechocystis* sp. PCC 6803	*ziaA*	40-fold	RT-PCR	Peca et al., 2007
*ziaA*	*Synechocystis* sp. PCC 6803	Inducer Zn^2+^ 4 μ M	*Synechocystis* sp. PCC 6803	*ziaA*	40-fold	RT-PCR	Blasi et al., 2012
*coat*	*Synechocystis* sp. PCC 6803	Inducer Zn^2+^ 3.2 μ M	*Synechocystis* sp. PCC 6803	*coaR + luxAB*	25-fold	25 RLU^e^	Peca et al., 2008
*coat*	*Synechocystis* sp. PCC 6803	Inducer Zn^2+^ 5 μ M	*Synechocystis* sp. PCC 6803	*coaT*	10-fold	RT-PCR	Peca et al., 2007
*coat*	*Synechocystis* sp. PCC 6803	Inducer Zn^2+^ 4 μ M	*Synechocystis* sp. PCC 6803	*coaT*	8-fold	RT-PCR	Blasi et al., 2012
*Smt*	*Synechococcus elongatus* PCC 7002	Inducer Zn^2+^ 2 μ M	*Synechocystis* sp. PCC 6803	Gene encoding EFE from *Pseudomonas syringae*	2-fold	2 nL ethylene mL^−1^ h^−1^	Guerrero et al., 2012
*ziaA^a^*	*Synechocystis* sp. PCC 6803	Inducer Zn^2+^ 3.5 μ M	*Synechocystis* sp. PCC 6803	*hydA1* from *Chlamydomonas reinhardtii*	Qualified but not quantified	109 nmol H_2_ mg Chl^−1^ min^−1^	Berto et al., 2011
**METABOLITE-INDUCIBLE PROMOTERS**							
*tetR^b^*	*E. coli*	Inducer aTc 10^3^ ng/per ml	*Synechocystis* sp. strain ATCC27184	*eYFP*	290-fold	>10,000 RFU	Huang and Lindblad, 2013
*trp-lac*	*E. coli*	Inducer IPTG 100 μ M	*Synechococcus elongatus* PCC 7942	*invA* and *glf* genes from *Zymomonas mobilis*	160-fold for fructose + 30-fold for glucose	160 μM fructose + 30 μM glucose	Niederholtmeyer et al., 2010
*Trc*	*E. coli*	Inducer IPTG 1 mM	*Synechococcus elongatus* PCC 7942	*uidA* from *E. coli*	36-fold	340 nmol MU min^−1^ mg protein^−1^ (β-Glucuronidase activity)	Geerts et al., 1995
*A1lacO-1*	*E. coli*	Inducer IPTG 1 mM	*Synechocystis* sp. PCC 6803	Gene encoding EFE from *Pseudomonas syringae*	8-fold	170 nL ethylene mL^−1^ h^−1^	Guerrero et al., 2012
*trc20*	*E. coli*	Inducer IPTG 2 mM	*Synechocystis* sp. PCC 6803	Gene encoding GFPmut3B	4-fold	12 RFU^e^ (relative to P*lacI*)	Huang et al., 2010
*trc10*	*E. coli*	Inducer IPTG 2 mM	*Synechocystis* sp. PCC 6803	Gene encoding GFPmut3B	1.6-fold	101 RFU^e^ (relative to PlacI)	Huang et al., 2010
*LlacO1^c^*	*E. coli*	Inducer IPTG 1 mM	*Synechococcus elongatus* PCC7942	*alsS (B. subtilis), alsD (A. hydrophila)*, and *adh (C. beijerinckii)*	1.6-fold	1.6 (relative to sADH and ALS activity)	Oliver et al., 2013
*Trc^d^*	*E. coli*	Inducer IPTG 1 mM	*Synechocystis* sp. PCC 6803	gene encoding EFE from *Pseudomonas syringae*	No significant difference	170 nL ethylene mL^−1^ h^−1^	Guerrero et al., 2012
**MACRONUTRIENT-INDUCIBLE PROMOTERS**							
*psbA2*	*Synechocystis* sp. PCC6803	Inducer light 500 μmol photons m^−2^ s^−1^	*Synechocystis* sp. PCC6803	*ispS* from *Pueraria montana* (kudzu)	Qualified but not quantified	~50 mg isoprene g DCW^−1^ d^−1^	Lindberg et al., 2010
*psbA2^a^*	*Synechocystis* sp. PCC 6803	Inducer light 50 μ Em^−2^s^−1^	*Synechocystis* sp. PCC 6803	*hydA1* from *Chlamydomonas reinhardtii*	Qualified but not quantified	130 nmol H_2_ mg Chl^−1^ min^−1^	Berto et al., 2011
*psbA1*	*Anabaena* sp. PCC 7120	Inducer light 30 μ Em^−2^s^−1^	*Anabaena* sp. PCC 7120	*hetR* from *E. coli*		17% heterocyst frequency	Chaurasia and Apte, 2011
*nirA*	*Synechococcus elongatus* PCC 7942	Inducer/Repressor NO^−^_3_/NH^+^_4_ 17.6 mM/17.6 mM	*Synechocystis* sp. PCC 6803	Gene encoding p-hydroxyphenylpyruvate dioxygenase from *Arabidopsis thaliana*	25-fold	250 ng tocopherol mg DCW^−1^	Qi et al., 2005
*nirA*	*Synechococcus elongatus* PCC 7942	Inducer/Repressor NO^−^_3_/NH^+^_4_ 15.0 mM/3.75 mM	*Synechococcus elongatus* PCC 7942	*cmpABCD*	5-fold	260 nmol HCO^−^_3_ mg Chl^−1^	Omata et al., 1999
*Nir*	*Anabaena* sp. PCC 7120	Inducer/Repressor NO^−^_3_/NH^+^_4_ 5.9 mM/10.0 mM	*Anabaena* sp. PCC 7120	*nir*	Qualified but not quantified	250 mg labeled proteins for NMR L^−1^	Desplancq et al., 2005

a In the presence of 5 μ M DCMU, which inhibits the PSII-dependent oxygen evolution.

b Grown in the dark on 5 mM glucose.

c Leaky production of 2,3-butanediol, no IPTG, and 1 mM IPTG similar.

d Plac variants had differential expression early in growth phase but dynamic range was reduced as growth proceeded.

e RFU = Relative Fluorescence Units; RLU = Relative Luminescence Units.

While the sensitivity of these promoters to low concentrations of ions may seem like an advantage, in practice it can make them difficult to use. Glassware must be thoroughly cleaned according to special protocols to remove trace metals and cells often have to be starved for extended periods, inducing stress responses, to use such inducible systems. Additionally, promoters endogenous to a chassis strain are woven into a complex, incompletely understood regulatory system. In this system, promoters are activated by multiple inducers, such as *PcoaT* (Co^2+^ and Zn^2+^) and *PziaA* (Cd^2+^ and Zn^2+^), both from *Synechocystis* sp. PCC 6803 and inducers can also activate multiple promoters, such as Cd^2+^ inducing *ziaA* and *isiA* (Blasi et al., 2012). Thus, these promoters fall short according to criteria 2, 3, and 5 described above.

While few good choices have so far been available for inducible promoters in cyanobacteria, it will be helpful to understand the differences in the cellular machinery of *E. coli* and cyanobacteria in order to adapt existing systems for use in a cyanobacterial “green *E. coli*.” First, RNA polymerase (RNAP) is structurally different between *E. coli* and cyanobacteria. In cyanobacteria the β ' subunit of the RNAP holoenzyme is split into two parts, as opposed to one in most eubacteria, creating a different DNA binding domain (Imamura and Asayama, 2009). Being photosynthetic, circadian, and sometimes nitrogen-fixing, cyanobacteria also employ three sets of interconnected σ factors that are different than those used by *E. coli* (Imamura and Asayama, 2009). Guerrero et al. (2012) looked at the variation in the −35 and −10 regions of P_A1lacO−1_ and P_trc_. P_trc_ is not inducible in *Synechocystis* sp PCC 6803 and had the “standard” bacterial structure in these regions while P_A1lacO−1_, which produced an 8-fold response to IPTG in the same host, had a different structure in both regions. They postulated that *Synechocystis* 6803′s sigma factors had different selectivity for these two regions. In fact, by systematically altering the bases between −10 and the transcription start site, a library of TetR-regulated promoters with improved inducibility were created in *Synechocystis* sp. strain ATCC27184 (a glucose-tolerant derivative of *Synechocystis* 6803). The best performing promoter induced a 290-fold change in response to 1 μg/ml aTc (Huang and Lindblad, 2013). This work demonstrates the improvements that can be seen when modifying parts to work in a particular chassis. However, the light-sensitivity of the inducer aTc required the use of special growth lights that may have had other effects on photoautotrophic metabolism. Further studies that follow in this vein of using well-characterized synthetic biology parts and modifying them to function optimally in a particular cyanobacterial chassis are likely to bear fruit.

The lack of inducibility seen in lac-derived promoters in cyanobacteria could also be a function of inadequate transport of IPTG into cells. Concentrations of IPTG above 1 mM have been shown to induce lac-derived promoters in organisms without an active lactose permease, like many cyanobacteria. By introducing an active lactose permease into *Pseudomonas fluorescens*, inducibility was boosted five times at 0.1 mM IPTG (Hansen et al., 1998). Evolving the Lac repressor for improved inducibility is another strategy. Gene expression improved ten times with 1 μ M IPTG through rounds of error prone PCR and DNA shuffling (Satya Lakshmi and Rao, 2009). Strength of expression and inducibility may also vary between different cyanobacterial strains. IPTG caused as much as a 36-fold response using the *trc* promoter in *Synechococcus elongatus* PCC 7942, but little or no response in *Synechocystis* sp. PCC 6803 (See Table 2). Phylogenetic analysis of σ factors from six different cyanobacterial strains, including *Synechocystis* 6803, showed *S. elongatus* 7942 to be distinctive. *S. elongatus* 7942 has σ factors that are unique to marine cyanobacteria as well as a group 3 σ factor similar to those from the heterocyst-forming *Anabaena* sp. PCC 7120 (Imamura and Asayama, 2009). Understanding these strain-specific differences will enhance the synthetic biologist's ability to design promoters with ideal characteristics in their chassis of choice. This relates to the ability to take up inducers as well as the optimal characteristics of inducers (as in the light-sensitivity of aTc) as described above.

### Reporters

Characterization of synthetic biological circuits depends on a reporting method to track the expression, interaction, and position of proteins. Preferably the reporter should be detected without destruction of the organisms or additional inputs. Bacterial luciferase and fluorescent proteins are the most common non-invasive reporters. The *lux* operon is frequently used for reporting in cyanobacteria (Michel et al., 2001; Mackey et al., 2007; Peca et al., 2008) and is well-suited for real time reporting of gene expression due to the short half-life of the relevant enzymes (Ghim et al., 2010). The superior brightness of fluorescent proteins makes them more ideal for subcellular localization via microscopy or for cell-sorting methods. Fluorescent proteins are produced in an array of colors and also do not require additional substrates. Their use in cyanobacteria is somewhat complicated by the fluorescence of the organism's photosynthetic pigments, but Cerulean, GFPmut3B (a mutant of green fluorescent protein) and EYFP (enhanced yellow fluorescent protein) have all been used successfully in cyanobacteria as reporters of gene expression (Huang et al., 2010; Heidorn et al., 2011; Landry et al., 2012; Huang and Lindblad, 2013).

Bacterial luciferase luminesces upon oxidation of reduced flavin mononucleotide (Meighen, 1993). Fluorescent proteins also require oxygen to correctly-fold and fluoresce (Hansen et al., 2001). The light-dark cycle of nitrogen-fixing cyanobacteria provides temporal separation of the oxygen-sensitive nitrogenase from oxygen-evolving photosynthesis (Golden et al., 1997). During the dark cycle, respiration reduces intra-cellular oxygen levels so that nitrogenase can function. Therefore, neither bacterial luciferase nor traditional fluorescent proteins can likely be used to study cyanobacteria in their dark cycle or to report on synthetic biology systems that operate in these oxygen-depleted conditions. Using blue light photoreceptors from *Bacillus subtilis* and *Pseudomonas putida*, oxygen-independent flavin mononucleotide-binding florescent proteins have been devised (Drepper et al., 2007). With an excitation wavelength of 450 nm and an emission wavelength of 495 nm, they should perform well in cyanobacteria, although no data supporting this has been published yet. Functionality of these new fluorescent proteins was also improved by replacing a phenylalanine suspected of quenching with serine or threonine, resulting in a doubling of the brightness (Mukherjee et al., 2012). This expanding variety of easily readable reporter systems will be extremely valuable for cyanobacterial synthetic biology.

### Cultivation systems

To date, most synthetic biology and metabolic engineering work in cyanobacteria has been performed using simple, low-tech cultivation methods such as shake flasks or bubbling tubes grown under standard fluorescent light sources. Often, laboratory incubators have simply been retrofitted by the addition of fluorescent light sources available in home improvement stores. However, as light and CO_2_ are major nutrients for cyanobacteria, it is critical to properly standardize the inputs of these resources to reliably characterize biological parts. It is also critical to increase the throughput of cyanobacterial growth systems to be able to screen the large numbers of variants that can be generated by combinatorial methods, as is routinely performed by growing heterotrophic bacterial cultures in 96-well plate format. Growth of cyanobacteria in 6-well plates can be routinely performed in our lab and by others (Huang and Lindblad, 2013) along with 24-well plates (Simkovsky et al., 2012), but growth in 96-well plates is poor, limiting assay throughput and requiring more space in lighted chambers under consistent illumination, which is often a limitation. Simple, low-cost systems to reproducibly grow many cyanobacterial cultures in parallel are necessary.

## Genome-scale modeling and fluxomics of cyanobacteria

**Figure 3:**
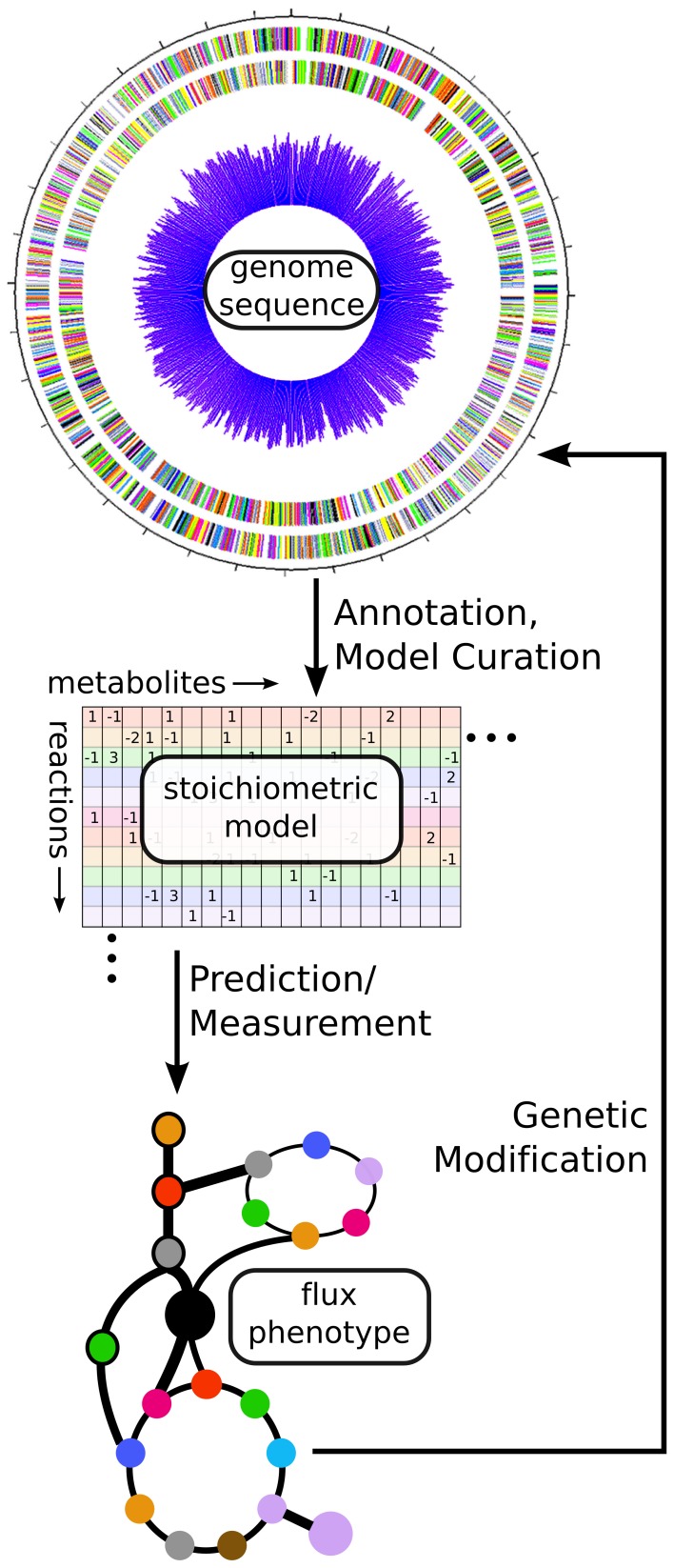
**Using fluxomics and genome scale models to link genotype to metabolic phenotype**. From an annotated genome sequence, a stoichiometric model of metabolism can be constructed. That model can be solved via either prediction of an optimal flux phenotype (FBA) or measurement of actual flux phenotype (^13^C-MFA). These results can help suggest modifications for altering the phenotype of the cell in a desired manner. In this way, a synthetic biologist can design new strains, build them using genetic modification methods, and test their phenotypes before designing new modifications in an iterative fashion.

A primary aim of cyanobacterial synthetic biology is the production of particular metabolites as biofuels or platform chemicals. As such, better understanding the metabolic phenotypes of wild-type and synthetic strains is a critical aim. While cyanobacterial metabolomics have been recently reviewed (Schwarz et al., 2013), here we describe recent progress in genome-scale modeling and fluxomics of cyanobacteria. These approaches can help guide the creation of synthetic strains with desirable metabolic phenotypes such as biofuel overproduction via *in silico* prediction or *in vivo* measurement of metabolic fluxes (See Figure 3). Specific to cyanobacterial systems, we highlight a number of challenges including complexity of modeling the photosynthetic metabolism and performing flux balance analysis (FBA), poor annotations of important metabolic pathways, and unavailability of *in vivo* gene essentiality information for most cyanobacteria. Finally, we focus on recent advancements in this area.

### Challenges

#### Incorporating photoautotrophy into metabolic models

FBA is a tool to make quantitative *in silico* predictions about metabolism (Fell and Small, 1986; Savinell and Palsson, 1992; Varma et al., 1993; Orth et al., 2010). An FBA model incorporates the stoichiometry of all genome-encoded metabolic reactions and assumes steady-state growth, such as during exponential phase. This assumption leads to a model that consists of a system of algebraic equations which state that the rate of producing any given metabolite is equal to the rate of consuming that metabolite. A solution to this system of equations is a possible answer to the question “what are all the metabolic fluxes in this system?” Since there are usually more reactions than metabolites, this system of equations is underdetermined and has many possible solutions. Therefore, one has to pick a solution that satisfies a biological objective, such as maximal growth, energy production, or byproduct formation (Varma and Palsson, 1994). For this purpose, a model will also include upper and lower bounds of fluxes that constrain the model to produce physically and biologically reasonable solutions.

Success of FBA greatly depends on the quality of the metabolic network reconstruction as well as the availability of regulatory constraints under a given environmental or growth condition. For instance, constraints can be added that disable or limit fluxes due to known regulatory constraints or substrate availability (Zomorrodi et al., 2012). For cyanobacteria, the major challenges to develop a genome-scale metabolic model and subsequently perform FBA are the same ones faced by these organisms in their diurnal environment: how to incorporate light and how to differentiate light and dark metabolisms. Although it has been nearly a decade since publication of the first study applying FBA to cyanobacteria, it is only recently that models have incorporated complete descriptions of the light reactions of photosynthesis (Nogales et al., 2012). In so doing, these authors were able to highlight the critical importance of alternate electron flow pathways to growth under diverse environmental conditions, and to identify differences in metabolism during carbon-limited and light-limited growth. However, debate remains among photosynthesis researchers about the exact form of the light reactions (Heyes and Hunter, 2005; Kopecna et al., 2013). This uncertainty about the exact stoichiometry of metabolism is a challenge for the predictive power of FBA in photosynthetic systems. While FBA requires the assumption of a pseudo-steady state, all cyanobacteria must alternate between day and night metabolisms during a diurnal cycle. A recent model (Saha et al., 2012) of *Cyanothece* sp. ATCC 51142 utilizes proteomic data to model the diurnal rhythm of this strain, which fixes carbon during the day and nitrogen during the night (see section Unique Challenges of the Cyanobacterial Lifestyle).

#### Incompleteness of genome annotation

Genome scale models are built starting with an annotated genome sequence (see Figure 3), which allows prediction of which metabolic reactions are available in a given strain. However, genome annotation is constantly evolving, and open questions remain about important metabolic reactions in cyanobacteria.

The understanding of several key pathways in cyanobacteria has been recently revised. Zhang and Bryant (2011) identified enzymes from *Synechococcus* 7002 that can complete the TCA cycle *in vitro* and have homologues in most cyanobacterial species, which were previously thought to possess an incomplete TCA cycle. Based on this information, *Synechocystis* 6803 model *i*Syn731 (Saha et al., 2012) allows for a complete TCA cycle including these reactions. However, using flux variability analysis (Mahadevan et al., 2002; Mahadevan and Schilling, 2003) it was determined that this alternate pathway is not essential for maximal biomass production [unpublished results, (Saha et al., 2012)]. Fatty acid metabolism in cyanobacteria has unique properties that have been recently uncovered due to increased interest in these pathways for biofuel production. Both *Synechocystis* sp. PCC 6803 and *Synechococcus elongatus* PCC 7942 contain a single candidate gene annotated for fatty acid activation. While in both organisms the gene is annotated as acyl-CoA synthetase, it shows only acyl-ACP synthetase activity instead (Kaczmarzyk and Fulda, 2010). Further analysis also shows the importance of acyl-ACP synthetase in enabling the transfer of fatty acids across the membrane (Von Berlepsch et al., 2012). Quinone synthesis is another pathway with conflicting annotations. Cyanobacteria contain neither ubiquinone nor menaquinone (Collins and Jones, 1981). Despite the lack of ubiquinone within cyanobacteria, a number of cyanobacterial genomes contain homologs for six *E. coli* genes involved in ubiquinone biosynthesis (Sakuragi, 2004). Given these homologous genes it is probable that plastoquinone, a quinone molecule participating in the electron transport chain, is produced in cyanobacteria using a pathway very similar to that of ubiquinone production in proteobacteria. Wu et al. (2010) showed that *Cyanothece* 51142 contains an alternative pathway for isoleucine biosynthesis. Threonine ammonia-lyase, catalyzing the conversion of threonine to 2-ketobutyrate, is absent in *Cyanothece* 51142. Instead, this organism uses a citramalate pathway with pyruvate and acetyl-CoA as precursors for isoleucine synthesis. An intermediate in this pathway, namely ketobutyrate, can be converted to higher alcohols (propanol and butanol) via this non-fermentative alcohol production pathway. These active areas of research will help to better define cyanobacterial metabolism and allow the generation of models that can more accurately predict cellular phenotypes. While newer fluxomics techniques can yield powerful results in well-characterized strains, developing a “green *E. coli*” will also require expanded knowledge of biochemistry that to date can only come from older methods of single gene or single protein analysis.

#### Fewer mutant resources to test model accuracy

The quality or accuracy of any genome-scale metabolic model can be tested by contrasting the *in silico* growth phenotype with available experimental data on the viability of single or multiple gene knockouts (Thiele and Palsson, 2010). Any discrepancies between model predictions and observed results can aid in model refinement (Kumar and Maranas, 2009). For model strains besies cyanobacteria, concerted efforts to create complete mutant libraries have led to improvements in metabolic modeling. To the best of our knowledge, extensive *in vivo* gene essentiality data are available only for *Synechocystis* 6803 among the cyanobacteria in the CyanoMutants database (Nakamura et al., 1999; Nakao et al., 2010), but only for ~119 genes, compared with 731 genes associated with metabolic reactions in a recent genome-scale model (Saha et al., 2012). Thus, only a small subset of the model predictions on gene essentiality can be evaluated using available data for *Synechocystis* 6803, and the proportion is much less for any other strain. While a genome-wide library of knockout mutants has been created in *Synechococcus* 7942 (Chen et al., 2012) segregation (and thus essentiality) has only been checked for a small selection of these mutants and its not available in any large-scale public database to date. Unavailability of such mutant information limits model validation and in turn hurts the value of computational predictions from FBA. Efforts to create complete mutant libraries in model cyanobacterial strains would improve the fidelity of genome-scale metabolic models, leading to testable hypotheses about how to alter metabolism for metabolite overproduction.

### Recent advances

#### Detailed genome-scale models

Genome-scale models contain detailed Gene-Protein-Reaction associations, a stoichiometric representation of all possible reactions occurring in an organism, and a set of appropriate regulatory constraints on each reaction flux. They are differentiated from more basic FBA models simply by their completeness—they span all or nearly all of the metabolic reactions encoded in a genome. Thus, these models can have greater predictive value than those of only central metabolism. Among cyanobacteria, *Cyanothece* 51142 exhibits one of the highest rates of nitrogen fixation and is the first unicellular diazotrophic cyanobacterium to be completely sequenced (Welsh et al., 2008). The first genome-scale model for *Cyanothece* 51142, *i*Cce806, is recently developed (Vu et al., 2012), while another more recent genome-scale model *i*Cyt773 contains an additional 266 unique reactions spanning pathways such as lipid, pigment and alkane biosynthesis (Saha et al., 2012). *i*Cyt773 also models diurnal metabolism by including flux regulation based on available day/night protein expression data (Stockel et al., 2011) and developing separate (light/dark) biomass equations. These models greatly enhance the ability to make computational predictions about this unique and promising diazotrophic organism.

Since *Synechocystis* 6803 is a model cyanobacterial strain, it has long been the target for modeling of photosynthetic central metabolism (Yang et al., 2002; Shastri and Morgan, 2005). More recent models (Knoop et al., 2010; Montagud et al., 2011) analyze growth under different conditions and detect bottlenecks and gene knock-out candidates to enhance metabolite production (e.g., ethanol, succinate, and hydrogen). A recent model represents the photosynthetic apparatus in detail, detects alternate flow pathways of electrons and also pinpoints photosynthetic robustness during photoautotrophic metabolism (Nogales et al., 2012). *i*Syn731, the latest of all *Synechocystis* 6803 models, integrates all recent developments and supplements them with improved metabolic capability and additional literature evidence. As many as 322 unique reactions are introduced in *i*Syn731 including reactions distributed in pathways such as heptadecane and fatty acid biosynthesis (Saha et al., 2012). Furthermore, *i*Syn731 is the first model for which both gene essentiality data (Nakamura et al., 1999) and MFA flux data (Young et al., 2011) are utilized to assess the predictive quality. Additionally, genome scale modeling has been extended to include another model cyanobacterium, *Synechococcus* sp. PCC 7002 (Hamilton and Reed, 2012). Other model strains highlighted in Table 1 have not yet had genome-scale models generated for their metabolism. Thus, stoichiometric models are emerging as a valuable tool for use across model cyanobacterial systems.

#### ^13^C MFA analysis

While *in silico* models are great tools for generating hypotheses on how to use synthetic biology interventions to alter metabolism, they need to be complemented by fluxomics methods that allow *in vivo* measurement of metabolic fluxes to assess these interventions. Such a suite of tools allows the closure of the design-build-test engineering cycle in synthetic biology (Figure 3). To this end, Young et al. (2011) have developed a method to measure fluxes in autotrophic metabolism via dynamic isotope labeling measurements. In this approach, cultures are fed with a step-change from naturally labeled bicarbonate to NaH^13^CO_3_ and the labeling patterns of metabolic intermediates are followed over a time-course to determine relative rates of metabolic flux. Previous studies (Yang et al., 2002) have also assessed metabolic fluxes under mixotrophic growth conditions, using a pseudo-steady-state approach in which cells are fed with ^13^C labeled glucose and metabolic fluxes are inferred from labeling patterns of proteinogenic amino acids. These studies have been extremely useful in identifying fluxes that exist *in vivo*, but have previously been regarded as wasteful or futile cycles, such as the oxidative pentose phosphate pathway and RuBP oxygenation. Comparisons between flux measurements (Young et al., 2011) and flux predictions (Saha et al., 2012) for *Synechocystis* 6803 have revealed the necessity of additional regulatory information for accurate *in silico* predictions of phenotype. These modeling and fluxomics efforts have resulted in deeper understanding of the metabolic capabilities of the modeled strains and of cyanobacteria in general.

## Conclusions

Cyanobacterial synthetic biology offers great promise for enhancing efforts to produce biofuels and chemicals in photoautotrophic hosts. While several cyanobacterial chassis strains have been used in synthetic biology efforts, the tools for their manipulation and analysis need greater development to unlock this potential and develop a “green *E. coli*.” Metabolic modeling is a complementary tool that can help guide the creation of synthetic strains with desirable phenotypes. By developing the tools for strain manipulation and control, synthetic biologists can unlock a bright future for the biotechnological use of abundant light and CO_2_.

### Conflict of interest statement

The authors declare that the research was conducted in the absence of any commercial or financial relationships that could be construed as a potential conflict of interest.

## References

[B1] Akiyama S. (2012). Structural and dynamic aspects of protein clocks: how can they be so slow and stable? Cell. Mol. Life Sci. 69, 2147–2160 10.1007/s00018-012-0919-3 22273739 PMC11114763

[B2] Ashby M. K. Houmard J. (2006). Cyanobacterial two-component proteins: structure, diversity, distribution, and evolution. Microbiol. Mol. Biol. Rev. 70, 472–509 10.1128/MMBR.00046-05 16760311 PMC1489541

[B3] Atsumi S. Higashide W. Liao J. C. (2009). Direct photosynthetic recycling of carbon dioxide to isobutyraldehyde. Nat. Biotechnol. 27, 1177–1180 10.1038/nbt.1586 19915552

[B4] Berla B. M. Pakrasi H. B. (2012). Upregulation of plasmid genes during stationary phase in *Synechocystis* sp. strain PCC 6803, a cyanobacterium. Appl. Environ. Microbiol. 78, 5448–5451 10.1128/AEM.01174-12 22636001 PMC3416404

[B5] Berto P. D'adamo S. Bergantino E. Vallese F. Giacometti G. M. Costantini P. (2011). The cyanobacterium *Synechocystis* sp. PCC 6803 is able to express an active [FeFe]-hydrogenase without additional maturation proteins. Biochem. Biophys. Res. Commun. 405, 678–683 10.1016/j.bbrc.2011.01.095 21284939

[B6] Blasi B. Peca L. Vass I. Kos P. B. (2012). Characterization of stress responses of heavy metal and metalloid inducible promoters in *Synechocystis* PCC6803. J. Microbiol. Biotechnol. 22, 166–169 10.4014/jmb.1106.06050 22370344

[B7] Boyanapalli R. Bullerjahn G. S. Pohl C. Croot P. L. Boyd P. W. McKay R. M. (2007). Luminescent whole-cell cyanobacterial bioreporter for measuring Fe availability in diverse marine environments. Appl. Environ. Microbiol. 73, 1019–1024 10.1128/AEM.01670-06 17158623 PMC1800772

[B8] Callahan S. M. Buikema W. J. (2001). The role of HetN in maintenance of the heterocyst pattern in *Anabaena* sp. PCC 7120. Mol. Microbiol. 40, 941–950 10.1046/j.1365-2958.2001.02437.x 11401701

[B9] Chaurasia A. K. Apte S. K. (2011). Improved eco-friendly recombinant *Anabaena* sp. strain PCC7120 with enhanced nitrogen biofertilizer potential. Appl. Environ. Microbiol. 77, 395–399 10.1128/AEM.01714-10 21057013 PMC3020549

[B10] Cheah Y. E. Albers S. C. Peebles C. A. (2013). A novel counter-selection method for markerless genetic modification in *Synechocystis* sp. PCC 6803. Biotechnol. Prog. 29, 23–30 10.1002/btpr.1661 23124993

[B11] Chen Y. Holtman C. K. Taton A. Golden S. S. (2012). Functional analysis of the synechococcus elongatus PCC 7942 Genome, in Functional Genomics and Evolution of Photosynthetic Systems, eds Burnap R. Vermaas W. (Springer), 119–137 Available online at: http://www.springer.com/life+sciences/book/978-94-007-1532-5

[B12] Collins M. D. Jones D. (1981). Distribution of isoprenoid quinone structural types in bacteria and their taxonomic implications. Microbiol. Rev. 45, 316–354 7022156 10.1128/mr.45.2.316-354.1981 PMC281511

[B13] Desplancq D. Bernard C. Sibler A. P. Kieffer B. Miguet L. Potier N. (2005). Combining inducible protein overexpression with NMR-grade triple isotope labeling in the cyanobacterium *Anabaena* sp. PCC 7120. Biotechniques 39, 405–411 10.2144/05393RR02 16206912

[B14] Drepper T. Eggert T. Circolone F. Heck A. Krauss U. Guterl J. K. (2007). Reporter proteins for *in vivo* fluorescence without oxygen. Nat. Biotechnol. 25, 443–445 10.1038/nbt1293 17351616

[B15] Duhring U. Axmann I. M. Hess W. R. Wilde A. (2006). An internal antisense RNA regulates expression of the photosynthesis gene *isiA*. Proc. Natl. Acad. Sci. U.S.A. 103, 7054–7058 10.1073/pnas.0600927103 16636284 PMC1459017

[B16] Dunlop M. J. Dossani Z. Y. Szmidt H. L. Chu H. C. Lee T. S. Keasling J. D. (2011). Engineering microbial biofuel tolerance and export using efflux pumps. Mol. Syst. Biol. 7, 487 10.1038/msb.2011.21 21556065 PMC3130554

[B17] Engler C. Marillonnet S. (2011). Generation of families of construct variants using golden gate shuffling. Methods Mol. Biol. 729, 167–181 10.1007/978-1-61779-065-2_11 21365490

[B18] Erbe J. L. Adams A. C. Taylor K. B. Hall L. M. (1996). Cyanobacteria carrying an smt-lux transcriptional fusion as biosensors for the detection of heavy metal cations. J. Ind. Microbiol. 17, 80–83 10.1007/BF01570047 8987894

[B19] Fell D. A. Small J. R. (1986). Fat synthesis in adipose tissue. An examination of stoichiometric constraints. Biochem. J. 238, 781–786 3800960 10.1042/bj2380781 PMC1147204

[B20] Geerts D. Bovy A. De Vrieze G. Borrias M. Weisbeek P. (1995). Inducible expression of heterologous genes targeted to a chromosomal platform in the cyanobacterium *Synechococcus* sp. PCC 7942. Microbiology 141(Pt 4), 831–841 10.1099/13500872-141-4-831 7773387

[B21] Ghim C. M. Lee S. K. Takayama S. Mitchell R. J. (2010). The art of reporter proteins in science: past, present and future applications. BMB Rep. 43, 451–460 10.5483/BMBRep.2010.43.7.451 20663405

[B22] Gibson D. G. Young L. Chuang R. Y. Venter J. C. Hutchison C. A. 3rd. Smith H. O. (2009). Enzymatic assembly of DNA molecules up to several hundred kilobases. Nat. Methods 6, 343–345 10.1038/nmeth.1318 19363495

[B23] Gierga G. Voss B. Hess W. R. (2009). The Yfr2 ncRNA family, a group of abundant RNA molecules widely conserved in cyanobacteria. RNA Biol. 6, 222–227 10.4161/rna.6.3.8921 19502815

[B24] Golden S. S. Ishiura M. Johnson C. H. Kondo T. (1997). Cyanobacterial Circadian Rhythms. Annu. Rev. Plant Physiol. Plant Mol. Biol. 48, 327–354 10.1146/annurev.arplant.48.1.327 15012266

[B25] Gronenberg L. S. Marcheschi R. J. Liao J. C. (2013). Next generation biofuel engineering in prokaryotes. Curr. Opin. Chem. Biol. 10.1016/j.cbpa.2013.03.037 23623045 PMC4211605

[B26] Guerrero F. Carbonell V. Cossu M. Correddu D. Jones P. R. (2012). Ethylene synthesis and regulated expression of recombinant protein in *Synechocystis* sp. PCC 6803. PLoS ONE 7:e50470 10.1371/journal.pone.0050470 23185630 PMC3503970

[B27] Hamilton J. J. Reed J. L. (2012). Identification of functional differences in metabolic networks using comparative genomics and constraint-based models. PLoS ONE 7:e34670 10.1371/journal.pone.0034670 22666308 PMC3359066

[B28] Hansen L. H. Knudsen S. Sorensen S. J. (1998). The effect of the lacY gene on the induction of IPTG inducible promoters, studied in Escherichia coli and Pseudomonas fluorescens. Curr. Microbiol. 36, 341–347 10.1007/s002849900320 9608745

[B29] Hansen M. C. Palmer R. J. Jr. Udsen C. White D. C. Molin S. (2001). Assessment of GFP fluorescence in cells of *Streptococcus gordonii* under conditions of low pH and low oxygen concentration. Microbiology 147, 1383–1391 11320140 10.1099/00221287-147-5-1383

[B30] Heidorn T. Camsund D. Huang H. H. Lindberg P. Oliveira P. Stensjo K. (2011). Synthetic biology in cyanobacteria engineering and analyzing novel functions. Meth. Enzymol. 497, 539–579 10.1016/B978-0-12-385075-1.00024-X 21601103

[B31] Heyes D. J. Hunter C. N. (2005). Making light work of enzyme catalysis: protochlorophyllide oxidoreductase. Trends Biochem. Sci. 30, 642–649 10.1016/j.tibs.2005.09.001 16182531

[B32] Higa K. C. Callahan S. M. (2010). Ectopic expression of hetP can partially bypass the need for hetR in heterocyst differentiation by *Anabaena* sp. strain PCC 7120. Mol. Microbiol. 77, 562–574 10.1111/j.1365-2958.2010.07257.x 20545862

[B33] Hilson N. Rosengarten R. Keasling J. (2012). j5 DNA assembly design automation software. ACS Synth. Biol. 1, 14–21 10.1021/sb2000116 23651006

[B34] Holtman C. Chen Y. Sandoval P. Gonzales A. Nalty M. Thomas T. (2005). High-throughput functional analysis of the *Synechococcus elongatus* PCC 7942 Genome. DNA Res. 12, 103–115 10.1093/dnares/12.2.103 16303742

[B35] Huang H. H. Camsund D. Lindblad P. Heidorn T. (2010). Design and characterization of molecular tools for a Synthetic Biology approach towards developing cyanobacterial biotechnology. Nucleic Acids Res. 38, 2577–2593 10.1093/nar/gkq164 20236988 PMC2860132

[B36] Huang H. H. Lindblad P. (2013). Wide-dynamic-range promoters engineered for cyanobacteria. J. Biol. Eng. 7:10 10.1186/1754-1611-7-10 23607865 PMC3724501

[B37] Imamura S. Asayama M. (2009). Sigma factors for cyanobacterial transcription. Gene Regul. Syst. Bio. 3, 65–87 19838335 10.4137/grsb.s2090 PMC2758279

[B38] Jones K. L. Kim S. W. Keasling J. D. (2000). Low-copy plasmids can perform as well as or better than high-copy plasmids for metabolic engineering of bacteria. Metab. Eng. 2, 328–338 10.1006/mben.2000.0161 11120644

[B39] Kaczmarzyk D. Fulda M. (2010). Fatty acid activation in cyanobacteria mediated by acyl-acyl carrier protein synthetase enables fatty acid recycling. Plant Physiol. 152, 1598–1610 10.1104/pp.109.148007 20061450 PMC2832271

[B40] Knoop H. Zilliges Y. Lockau W. Steuer R. (2010). The metabolic network of *Synechocystis* sp. PCC 6803: systemic properties of autotrophic growth. Plant Physiol. 154, 410–422 10.1104/pp.110.157198 20616194 PMC2938163

[B41] Kopecna J. Sobotka R. Komenda J. (2013). Inhibition of chlorophyll biosynthesis at the protochlorophyllide reduction step results in the parallel depletion of Photosystem I and Photosystem II in the cyanobacterium *Synechocystis* PCC 6803. Planta 237, 497–508 10.1007/s00425-012-1761-4 23011568

[B42] Kumar V. S. Maranas C. D. (2009). GrowMatch: an automated method for reconciling *in silico/in vivo* growth predictions. PLoS Comput. Biol. 5:e1000308 10.1371/journal.pcbi.1000308 19282964 PMC2645679

[B43] Kunert A. Vinnemeier J. Erdmann N. Hagemann M. (2003). Repression by Fur is not the main mechanism controlling the iron-inducible isiAB operon in the cyanobacterium *Synechocystis* sp. PCC 6803. FEMS Microbiol. Lett. 227, 255–262 10.1016/S0378-1097(03)00689-X 14592717

[B44] Lagarde D. Beuf L. Vermaas W. (2000). Increased production of zeaxanthin and other pigments by application of genetic engineering techniques to *Synechocystis* sp. Strain PCC 6803. Appl. Environ. Microbiol. 66, 64–72 10.1128/AEM.66.1.64-72.2000 10618204 PMC91786

[B45] Landry B. Stockel J. Pakrasi H. (2012). Use of degradation tags to control protein levels in the cyanobacterium *Synechocystis* sp. Strain PCC 6803. Appl. Environ. Microbiol. 70, 2833–2835 23396339 10.1128/AEM.03741-12 PMC3623166

[B46] Latysheva N. Junker V. L. Palmer W. J. Codd G. A. Barker D. (2012). The evolution of nitrogen fixation in cyanobacteria. Bioinformatics 28, 603–606 10.1093/bioinformatics/bts008 22238262

[B47] Li M. Z. Elledge S. J. (2007). Harnessing homologous recombination *in vitro* to generate recombinant DNA via SLIC. Nat. Methods 4, 251–256 10.1038/nmeth1010 17293868

[B48] Lindberg P. Park S. Melis A. (2010). Engineering a platform for photosynthetic isoprene production in cyanobacteria, using *Synechocystis* as the model organism. Metab. Eng. 12, 70–79 10.1016/j.ymben.2009.10.001 19833224

[B49] Liu X. Sheng J. Curtiss R. 3rd. (2011). Fatty acid production in genetically modified cyanobacteria. Proc. Natl. Acad. Sci. U.S.A. 108, 6899–6904 10.1073/pnas.1103014108 21482809 PMC3084101

[B50] Mackey S. R. Ditty J. L. Clerico E. M. Golden S. S. (2007). Detection of rhythmic bioluminescence from luciferase reporters in cyanobacteria. Methods Mol. Biol. 362, 115–129 10.1007/978-1-59745-257-1_8 17417005

[B51] Mahadevan R. Edwards J. S. Doyle F. J. (2002). Dynamic flux balance analysis of diauxic growth in Escherichia coli. Biophys. J. 83, 1331–1340 10.1016/S0006-3495(02)73903-9 12202358 PMC1302231

[B52] Mahadevan R. Schilling C. H. (2003). The effects of alternate optimal solutions in constraint-based genome-scale metabolic models. Metab. Eng. 5, 264–276 10.1016/j.ymben.2003.09.002 14642354

[B53] Meighen E. A. (1993). Bacterial bioluminescence: organization, regulation, and application of the lux genes. FASEB J. 7, 1016–1022 8370470 10.1096/fasebj.7.11.8370470

[B54] Melis A. (2013). Carbon partitioning in photosynthesis. Curr. Opin. Chem. Biol. 17, 453–456 10.1016/j.cbpa.2013.03.010 23542013

[B55] Michel K. P. Pistorius E. K. Golden S. S. (2001). Unusual regulatory elements for iron deficiency induction of the *idiA* gene of *Synechococcus elongatus* PCC 7942. J. Bacteriol. 183, 5015–5024 10.1128/JB.183.17.5015-5024.2001 11489854 PMC95377

[B56] Mitschke J. Georg J. Scholz I. Sharma C. M. Dienst D. Bantscheff J. (2011). An experimentally anchored map of transcriptional start sites in the model cyanobacterium *Synechocystis* sp. PCC6803. Proc. Natl. Acad. Sci. U.S.A. 108, 2124–2129 10.1073/pnas.1015154108 21245330 PMC3033270

[B57] Montagud A. Zelezniak A. Navarro E. De Cordoba P. Urchueguia J. F. Patil K. R. (2011). Flux coupling and transcriptional regulation within the metabolic network of the photosynthetic bacterium *Synechocystis* sp PCC6803. Biotechnol. J. 6, 330–342 10.1002/biot.201000109 21226012

[B58] Montgomery B. L. (2007). Sensing the light: photoreceptive systems and signal transduction in cyanobacteria. Mol. Microbiol. 64, 16–27 10.1111/j.1365-2958.2007.05622.x 17376068

[B59] Moon T. S. Lou C. Tamsir A. Stanton B. C. Voigt C. A. (2012). Genetic programs constructed from layered logic gates in single cells. Nature 491, 249–253 10.1038/nature11516 23041931 PMC3904217

[B60] Mukherjee A. Weyant K. B. Walker J. Schroeder C. M. (2012). Directed evolution of bright mutants of an oxygen-independent flavin-binding fluorescent protein from *Pseudomonas putida*. J. Biol. Eng. 6, 20 10.1186/1754-1611-6-20 23095243 PMC3488000

[B61] Nagarajan A. Winter R. Eaton-Rye J. Burnap R. (2011). A synthetic DNA and fusion PCR approach to the ectopic expression of high levels of the D1 protein of photosystem II in *Synechocystis* sp. PCC 6803. J. Photochem.Photobiol. B 104, 212–219 10.1016/j.jphotobiol.2011.02.009 21377372

[B62] Nakajima M. Imai K. Ito H. Nishiwaki T. Murayama Y. Iwasaki H. (2005). Reconstitution of circadian oscillation of cyanobacterial KaiC phosphorylation *in vitro*. Science 308, 414–415 10.1126/science.1108451 15831759

[B63] Nakamura Y. Kaneko T. Miyajima N. Tabata S. (1999). Extension of CyanoBase. CyanoMutants: repository of mutant information on *Synechocystis* sp. strain PCC6803. Nucleic Acids Res. 27, 66–68 10.1093/nar/27.1.66 9847144 PMC148099

[B64] Nakao M. Okamoto S. Kohara M. Fujishiro T. Fujisawa T. Sato S. (2010). CyanoBase: the cyanobacteria genome database update 2010. Nucleic Acids Res. 38, D379–D381 10.1093/nar/gkp915 19880388 PMC2808859

[B65] Ng W. O. Zentella R. Wang Y. Taylor J. S. Pakrasi H. B. (2000). PhrA, the major photoreactivating factor in the cyanobacterium *Synechocystis* sp. strain PCC 6803 codes for a cyclobutane-pyrimidine-dimer-specific DNA photolyase. Arch. Microbiol. 173, 412–417 10.1007/s002030000164 10896222

[B66] Niederholtmeyer H. Wolfstadter B. T. Savage D. F. Silver P. A. Way J. C. (2010). Engineering cyanobacteria to synthesize and export hydrophilic products. Appl. Environ. Microbiol. 76, 3462–3466 10.1128/AEM.00202-10 20363793 PMC2876443

[B67] Nogales J. Gudmundsson S. Knight E. M. Palsson B. O. Thiele I. (2012). Detailing the optimality of photosynthesis in cyanobacteria through systems biology analysis. Proc. Natl. Acad. Sci. U.S.A. 109, 2678–2683 10.1073/pnas.1117907109 22308420 PMC3289291

[B68] Oliver J. W. Machado I. M. Yoneda H. Atsumi S. (2013). Cyanobacterial conversion of carbon dioxide to 2,3-butanediol. Proc. Natl. Acad. Sci. U.S.A. 110, 1249–1254 10.1073/pnas.1213024110 23297225 PMC3557092

[B69] Omata T. Price G. D. Badger M. R. Okamura M. Gohta S. Ogawa T. (1999). Identification of an ATP-binding cassette transporter involved in bicarbonate uptake in the cyanobacterium *Synechococcus* sp. strain PCC 7942. Proc. Natl. Acad. Sci. U.S.A. 96, 13571–13576 10.1073/pnas.96.23.13571 10557362 PMC23989

[B70] Orth J. D. Thiele I. Palsson B. O. (2010). What is flux balance analysis? Nat. Biotechnol. 28, 245–248 10.1038/nbt.1614 20212490 PMC3108565

[B71] Peca L. Kos P. B. Mate Z. Farsang A. Vass I. (2008). Construction of bioluminescent cyanobacterial reporter strains for detection of nickel, cobalt and zinc. FEMS Microbiol. Lett. 289, 258–264 10.1111/j.1574-6968.2008.01393.x 19016871

[B72] Peca L. Kos P. B. Vass I. (2007). Characterization of the activity of heavy metal-responsive promoters in the cyanobacterium *Synechocystis* PCC 6803. Acta. Biol. Hung. 58, 11–22 10.1556/ABiol.58.2007.Suppl.2 18297791

[B73] Pfreundt U. Stal L. J. Voss B. Hess W. R. (2012). Dinitrogen fixation in a unicellular chlorophyll d-containing cyanobacterium. ISME J. 6, 1367–1377 10.1038/ismej.2011.199 22237545 PMC3379635

[B74] Qi Q. Hao M. Ng W. O. Slater S. C. Baszis S. R. Weiss J. D. (2005). Application of the *Synechococcus nirA* promoter to establish an inducible expression system for engineering the *Synechocystis* tocopherol pathway. Appl. Environ. Microbiol. 71, 5678–5684 10.1128/AEM.71.10.5678-5684.2005 16204475 PMC1265929

[B75] Quan J. Tian J. (2009). Circular polymerase extension cloning of complex gene libraries and pathways. PLoS ONE 4:e6441 10.1371/journal.pone.0006441 19649325 PMC2713398

[B76] Saha R. Verseput A. T. Berla B. M. Mueller T. J. Pakrasi H. B. Maranas C. D. (2012). Reconstruction and comparison of the metabolic potential of cyanobacteria *Cyanothece* sp. ATCC 51142 and *Synechocystis* sp. PCC 6803. PLoS ONE 7:e48285 10.1371/journal.pone.0048285 23133581 PMC3487460

[B77] Sakuragi Y. (2004). Studies of Quinones in Cyanobacteria. University Park, PA: Doctor of Philosophy; The Pennsylvania State University

[B78] Satya Lakshmi O. Rao N. M. (2009). Evolving Lac repressor for enhanced inducibility. Protein Eng. Des. Sel. 22, 53–58 10.1093/protein/gzn069 19029094

[B79] Savinell J. M. Palsson B. O. (1992). Network analysis of intermediary metabolism using linear optimization. I. Development of mathematical formalism. J. Theor. Biol. 154, 421–454 10.1016/S0022-5193(05)80161-4 1593896

[B80] Schwarz D. Orf I. Kopka J. Hagemann M. (2013). Recent applications of metabolomics toward cyanobacteria. Metabolites 3, 72–100 10.3390/metabo3010072 PMC3901253 24957891

[B81] Selinger D. W. Cheung K. J. Mei R. Johansson E. M. Richmond C. S. Blattner F. R. (2000). RNA expression analysis using a 30 base pair resolution *Escherichia coli* genome array. Nat. Biotechnol. 18, 1262–1268 10.1038/82367 11101804

[B82] Shao Z. Zhao H. (2009). DNA assembler, an *in vivo* genetic method for rapid construction of biochemical pathways. Nucleic Acids Res. 37:e16 10.1093/nar/gkn991 19074487 PMC2632897

[B83] Sharma C. M. Hoffmann S. Darfeuille F. Reignier J. Findeiss S. Sittka A. (2010). The primary transcriptome of the major human pathogen *Helicobacter pylori*. Nature 464, 250–255 10.1038/nature08756 20164839

[B84] Shastri A. A. Morgan J. A. (2005). Flux balance analysis of photoautotrophic metabolism. Biotechnol. Prog. 21, 1617–1626 10.1021/bp050246d 16321043

[B85] Simkovsky R. Daniels E. F. Tang K. Huynh S. C. Golden S. S. Brahamsha B. (2012). Impairment of O-antigen production confers resistance to grazing in a model amoeba-cyanobacterium predator-prey system. Proc. Natl. Acad. Sci. U.S.A. 109, 16678–16683 10.1073/pnas.1214904109 23012457 PMC3478625

[B86] Stockel J. Jacobs J. M. Elvitigala T. R. Liberton M. Welsh E. A. Polpitiya A. D. (2011). Diurnal rhythms result in significant changes in the cellular protein complement in the cyanobacterium *Cyanothece* 51142. PLoS ONE 6:e16680 10.1371/journal.pone.0016680 21364985 PMC3043056

[B87] Stockel J. Welsh E. A. Liberton M. Kunnvakkam R. Aurora R. Pakrasi H. B. (2008). Global transcriptomic analysis of *Cyanothece* 51142 reveals robust diurnal oscillation of central metabolic processes. Proc. Natl. Acad. Sci. U.S.A. 105, 6156–6161 10.1073/pnas.0711068105 18427117 PMC2329701

[B88] Szewczyk E. Nayak T. Oakley C. E. Edgerton H. Xiong Y. Taheri-Talesh N. (2007). Fusion PCR and gene targeting in Aspergillus nidulans. Nat. Protoc. 1, 3111–3120 10.1038/nprot.2006.405 17406574

[B89] Takahama K. Matsuoka M. Nagahama K. Ogawa T. (2004). High-frequency gene replacement in cyanobacteria using a heterologous rps12 gene. Plant Cell Physiol. 45, 333–339 10.1093/pcp/pch041 15047882

[B90] Tan X. Liang F. Cai K. Lu X. (2013). Application of the FLP/FRT recombination system in cyanobacteria for construction of markerless mutants. Appl. Microbiol. Biotechnol. 97, 6373–6382 10.1007/s00253-013-4837-6 23512480

[B91] Taniuchi Y. Yoshikawa S. Maeda S. Omata T. Ohki K. (2008). Diazotrophy under continuous light in a marine unicellular diazotrophic cyanobacterium, *Gloeothece* sp. 68DGA. Microbiology 154, 1859–1865 10.1099/mic.0.2008/018689-0 18599815

[B92] Taton A. Lis E. Adin D. M. Dong G. Cookson S. Kay S. A. (2012). Gene transfer in *Leptolyngbya* sp. strain BL0902, a cyanobacterium suitable for production of biomass and bioproducts. PLoS ONE 7:e30901 10.1371/journal.pone.0030901 22292073 PMC3265524

[B93] Teng S. W. Mukherji S. Moffitt J. R. De Buyl S. O'shea E. K. (2013). Robust circadian oscillations in growing cyanobacteria require transcriptional feedback. Science 340, 737–740 10.1126/science.1230996 23661759 PMC3696982

[B94] Thiele I. Palsson B. O. (2010). A protocol for generating a high-quality genome-scale metabolic reconstruction. Nat. Protoc. 5, 93–121 10.1038/nprot.2009.203 20057383 PMC3125167

[B95] Tyo K. E. Jin Y. S. Espinoza F. A. Stephanopoulos G. (2009). Identification of gene disruptions for increased poly-3-hydroxybutyrate accumulation in *Synechocystis* PCC 6803. Biotechnol. Prog. 25, 1236–1243 10.1002/btpr.228 19606467

[B96] Varma A. Boesch B. W. Palsson B. O. (1993). Stoichiometric interpretation of *Escherichia coli* glucose catabolism under various oxygenation rates. Appl. Environ. Microbiol. 59, 2465–2473 8368835 10.1128/aem.59.8.2465-2473.1993 PMC182307

[B97] Varma A. Palsson B. O. (1994). Stoichiometric flux balance models quantitatively predict growth and metabolic by-product secretion in wild-type *Escherichia coli* W3110. Appl. Environ. Microbiol. 60, 3724–3731 7986045 10.1128/aem.60.10.3724-3731.1994 PMC201879

[B98] Von Berlepsch S. Kunz H. H. Brodesser S. Fink P. Marin K. Flugge U. I. (2012). The acyl-acyl carrier protein synthetase from *Synechocystis* sp. PCC 6803 mediates fatty acid import. Plant Physiol. 159, 606–617 10.1104/pp.112.195263 22535424 PMC3375928

[B99] Vu T. T. Stolyar S. M. Pinchuk G. E. Hill E. A. Kucek L. A. Brown R. N. (2012). Genome-scale modeling of light-driven reductant partitioning and carbon fluxes in diazotrophic unicellular cyanobacterium *Cyanothece* sp. ATCC 51142. PLoS Comput. Biol. 8:e1002460 10.1371/journal.pcbi.1002460 22529767 PMC3329150

[B100] Wang B. Pugh S. Nielsen D. R. Zhang W. Meldrum D. R. (2013). Engineering cyanobacteria for photosynthetic production of 3-hydroxybutyrate directly from CO_2_. Metab. Eng. 16C, 68–77 10.1016/j.ymben.2013.01.001 23333586

[B101] Wang B. Wang J. Zhang W. Meldrum D. R. (2012). Application of synthetic biology in cyanobacteria and algae. Front. Microbiol. 3:344 10.3389/fmicb.2012.00344 23049529 PMC3446811

[B102] Waters C. M. Bassler B. L. (2006). The Vibrio harveyi quorum-sensing system uses shared regulatory components to discriminate between multiple autoinducers. Genes Dev. 20, 2754–2767 10.1101/gad.1466506 17015436 PMC1578700

[B103] Welsh E. A. Liberton M. Stockel J. Loh T. Elvitigala T. Wang C. (2008). The genome of *Cyanothece* 51142, a unicellular diazotrophic cyanobacterium important in the marine nitrogen cycle. Proc. Natl. Acad. Sci. U.S.A. 105, 15094–15099 10.1073/pnas.0805418105 18812508 PMC2567498

[B104] Woelfle M. A. Ouyang Y. Phanvijhitsiri K. Johnson C. H. (2004). The adaptive value of circadian clocks: an experimental assessment in cyanobacteria. Curr. Biol. 14, 1481–1486 10.1016/j.cub.2004.08.023 15324665

[B105] Wu B. Zhang B. C. Feng X. Y. Rubens J. R. Huang R. Hicks L. M. (2010). Alternative isoleucine synthesis pathway in cyanobacterial species. Microbiology 156, 596–602 10.1099/mic.0.031799-0 19875435

[B106] Xu Y. Alvey R. M. Byrne P. O. Graham J. E. Shen G. Bryant D. A. (2011). Expression of genes in cyanobacteria: adaptation of endogenous plasmids as platforms for high-level gene expression in *Synechococcus* sp. PCC 7002. Methods Mol. Biol. 684, 273–293 10.1007/978-1-60761-925-3_21 20960136

[B107] Xu Y. Ma P. Shah P. Rokas A. Liu Y. Johnson C. H. (2013). Non-optimal codon usage is a mechanism to achieve circadian clock conditionality. Nature 495, 116–120 10.1038/nature11942 23417065 PMC3593822

[B108] Yang C. Hua Q. Shimizu K. (2002). Metabolic flux analysis in *Synechocystis* using isotope distribution from 13C-labeled glucose. Metab. Eng. 4, 202–216 10.1006/mben.2002.0226 12616690

[B109] Young J. D. Shastri A. A. Stephanopoulos G. Morgan J. A. (2011). Mapping photoautotrophic metabolism with isotopically nonstationary (13)C flux analysis. Metab. Eng. 13, 656–665 10.1016/j.ymben.2011.08.002 21907300 PMC3210925

[B110] Zhang F. Carothers J. M. Keasling J. D. (2012). Design of a dynamic sensor-regulator system for production of chemicals and fuels derived from fatty acids. Nat. Biotechnol. 30, 354–359 10.1038/nbt.2149 22446695

[B111] Zhang S. Y. Bryant D. A. (2011). The tricarboxylic acid cycle in cyanobacteria. Science 334, 1551–1553 10.1126/science.1210858 22174252

[B112] Zhang Y. Pu H. Wang Q. Cheng S. Zhao W. Zhang Y. (2007). PII is important in regulation of nitrogen metabolism but not required for heterocyst formation in the Cyanobacterium *Anabaena* sp. PCC 7120. J. Biol. Chem. 282, 33641–33648 10.1074/jbc.M706500200 17875643

[B113] Zomorrodi A. R. Suthers P. F. Ranganathan S. Maranas C. D. (2012). Mathematical optimization applications in metabolic networks. Metab. Eng. 14, 672–686 10.1016/j.ymben.2012.09.005 23026121

